# From Our Correspondents

**Published:** 1984-10

**Authors:** 


### From Our Correspondents

**Permanent Exhibition of Medical History at Frenchay Hospital**

Forty years ago, many would have been forgiven for believing that most of Medical History had been written and it would have been impossible then to imagine that many additional chapters would have been documented by 1984 with the prospect of many more to come. In reality, many significant clinical developments have taken place even in the lifetime of still virile members of the health profession and there has of late been a resurgence of interest in Medical History as a worthwhile subject for research. Rather than being the prime interest of doctors at the end of a clinical lifetime of service, many active groups of Medical Historians exist throughout the country, and medical students are being encouraged to devote time in their elective periods to this subject. This is in great part due to the far-sightedness of the Wellcome Foundation and it is most appropriate that many are seeking today to preserve our wealth of Medical History.

Coincidentally with this renewed interest, it is with great pleasure that one can record for the first time anywhere in the provinces, the establishment of a permanent Exhibition Hall of Medical History. This has been made possible entirely due to the generosity of Mr. J. H. Britton, C.B.E., who has agreed to meet the building costs of such a project which is dedicated to the memory of his late wife Monica, who was well known for the service and tireless efforts she gave to many medical charities both in Bristol and beyond.

The Exhibition Hall will be a purpose built structure adjacent to the Post Graduate Centre at Frenchay Hospital; the building programme is well under way and should be completed by Spring 1985. The proposed development plans for Frenchay, the ease of access to the exhibition building with its distinctive design should do much to enhance its location and generate interest throughout the South West.

The aims and objects of the Exhibition Hall are to reflect the historical development of all aspects of medicine in general and to highlight those developments pioneered in the Bristol locality and throughout the South Western Region. There is no doubt that the South West has over the years played its part in the development of all branches of medicine and it is hoped that an exhibition will reflect such local and regional work, as well as that of a more general nature. Thus, there will be a fairly permanent display of exhibits as well as shorter term temporary displays designed to highlight anniversaries, specific functions, special events, etc. Lecture programmes will be organised and all those interested in Medical History will be catered for, including the general public.

It is anticipated that the Exhibition will function as a charity in an educational role. Not being in receipt of N.H.S. funding, it will rely heavily on donations and subscriptions from private organisations and individuals for its continued existence. The immediate concern is for the finance to provide for display cabinets and the day to day running and administration of the exhibits. There is no doubt of the unique nature of this project and it is hoped to attract the attention and interest of all in Bristol and throughout the South West.

In addition to finance, the Exhibition will depend upon donations or the loan of articles both from individuals and corporate bodies from all over the South West. So far, with modest publicity, many, both medical and the general public, have responded and exhibits have been received from as far afield as Sherborne and Barnstaple in addition to a major contribution from the Bristol hospitals, all of which are giving very strong support to this project. Further exhibits are required and if any reader has access to any item of equipment, interesting objects, medical memorabilia including documents, or anything they consider of interest which they would be prepared to donate or lend to the project, would they please contact Frenchay Postgraduate Centre.

It is also important to know of people in the region who have any special expertise or knowledge of medical history and who would be prepared to assist in the furtherance of the project.

All those working on the project are doing so in an honorary capacity although it is hoped to employ part-time staff in due course. A committee of management is shortly to be appointed together with the nomination of trustees and officers—and it is hoped that news of further developments will be made available through this Journal. In the meantime, all enquiries should be made to the Postgraduate Secretary, Frenchay Hospital, Bristol. Tel. 565656 ext 369.

J.A.B.

**Managers in the Health Service, Appointment in the South West Region**

Before the 1974 reorganisation of the Health Service there were individuals seen to have executive power and responsibility. For example, the Group Secretary was the individual generally seen to have such power in a Hospital Group. Since this reorganisation and the introduction of Management Teams responsibility has been blurred with the Administrator simply a member of a team. There has been a power vacuum. The Griffiths report has identified this defect in the administration of the N.H.S. and the recent introduction of Managers is designed to remedy this.

Managers have now been appointed to most of the Regional Health Authorities. They have been, so far, all officers already in service, though in theory anybody can be brought in from outside, e.g. from industry. Most of those appointed have been, not surprisingly, from the ranks of the administrators, but the Oxford Region has appointed its Regional Medical Officer, Dr. Rosemary Rue. The South West Region has elevated its Regional Nursing Officer, Miss Catherine Hawkins to be the Regional Manager. She still remains in charge of nursing. Formerly she was Chief Nursing Officer to the Southmead Health District and before that was at the Bristol Royal Infirmary.

Hitherto executive authority has not been made explicit and it is to be hoped that decisions will be clearer and made without delay. Those who seek decisions now know where to go for them and those who wish to apportion blame now know where to fix it. A new 'Hot Seat' has been created. Our Health Supremo in the South West has a formidable task but she has the reputation of being a formidable woman. We wish her well.

The next step is to appoint Managers to the Districts.

M.G.W.
W.J.A.

**Artificial Human Reproduction**

I have, over the past few weeks, been carrying out an informal poll. The subject has been the incidence in normal conversation, both in and out of the medical context, of the verbs 'can' and 'ought'. The results, though hardly scientific, are that I lost count of the number of times 'can' was used, but hardly reached double figures for 'ought' or 'should'. This suggests to me that, in 1984, people are more concerned with the technical question, 'Is something possible?' than the philosophical one, 'Should it be done?' This was particularly noticeable in the clinical context, a disturbing trend at a time when the recent rapid advances in medical technology seem to have provided all too fallible mankind with the capacity not merely to change the conditions of our human existence, but also its essential characteristics.

The techniques which tend to fall into this category and are the greatest cause of current debate are those involving artificial human reproduction—AID, IVF, surrogate motherhood, genetic engineering and the like. The Warnock Committee report (H.M.S.O., 1984) is a realistic and pragmatic attempt to deal with many of the basic problems. Although there was a moral philosophical and theological input into the deliberations of the committee, their remit was to try to achieve the difficult balance between the 'can' and the 'ought'. This, I believe, they did. However, we cannot (indeed dare not) assume that we as a profession or individuals are absolved from any further thought on the ethics of these issues. Unfortunately our training ill fits us for clear and logical ethical thought, and the preeminence of technology with which we have been inculcated means that, all too often, we seem to be moral pygmies.

I have recently been made all too aware of this by reading the paperback entitled 'Begotten or Made? Human Procreation and Medical Technique' by Oliver O'Donovan, Regius Professor of Moral and Pastoral Theology in the University of Oxford. Published by the Clarendon Press, Oxford, and costing only £2.50, it brought me up with a jolt in many areas of my thinking. I cannot say I agree with all his views, but it certainly made me put my own thinking under the microscope. I recommend it to you. Perhaps the next time I carry out my straw poll the 'oughts' will be closer to the 'cans'. I somehow doubt it, but if it were so, our patients and our students would be the beneficiaries.

G.M.S.

**Joe Peacock**

The last day of July saw the retirement of Professor J. H. Peacock after 36 years' service to Surgery in Bristol and the South West and on the national stage. Born in London, Joe had a mobile childhood. His father worked for the Great Western Railway and was promoted down the line. Joe Peacock was educated at Bristol Grammar School and was accepted to read Dentistry at Birmingham University, quickly changing to Medicine. After qualifying in 1941 he worked as House Surgeon at Wolverhampton Royal Hospital, where Mr. R. M. Walker was on the Surgical staff. He was called up in 1942 and spent 5 years with the R.A.M.C. in India and the Far East. Demobilised and unemployed he came to Bristol in 1948, where Robert Milnes Walker had recently taken the Chair. Apart from a year's research at Ann Arbor, Michigan, he never left the University Department of Surgery, being successively Supernumerary Registrar (1948), Senior Registrar (1950), Lecturer (1952), Consultant Surgeon (1955), Reader (1965) and Professor of Surgical Science (1969).

Joe's surgical interests have been catholic and his knowledge encyclopaedic, but he has achieved special prominence in the fields of vascular surgery and liver surgery. The vascular seeds were planted by Milnes Walker and the Rockefeller fellowship to the

U.S.A. in 1951. The pioneering work in liver transplantation was undertaken in collaboration with his friend, Professor Athol Riddell. The fifties and sixties were exciting and fruitful decades, marked by a series of honours from the Royal College of Surgeons: Hunterian Professorship, Arris and Gale Lectureship and the unique achievement of two Jacksonian Prizes. Joe Peacock examined in Primary F.R.C.S. from 1965 to 1970 and Final F.R.C.S. from 1976 to 1982, ending as Chairman of the Court of Examiners. A Doctorate of Medicine followed his Mastership of Surgery.

During the seventies and eighties Joe became increasingly saddled with administrative commitments. He joined both the South Western Regional Health Authority and the General Medical Council in 1974, being appointed Chairman of the G.M.C. Overseas Committee on Full Registration in 1980. Indeed, he still holds this onerous Chairmanship. Twice he was asked to assume at short notice the Headship of the University Department of Surgery, in 1974-75 and again in 1977–80. His contribution in providing smooth continuity on each occasion has sometimes been underrated but is particularly appreciated by this correspondent.

A celebratory dinner was held in honour of Professor Joe Peacock in the Merchant's Hall, Clifton, on 22nd June 1984. The 90 guests included the Vice-Chancellor and Lady Merrison, the President of the Royal College of Surgeons and Lady Slaney and a great company of friends and colleagues from Bristol and the South West. It was a happy and memorable occasion. The following day a Scientific meeting was held at the Infirmary. Two visiting Professors of Surgery, Norman Browse and Ken Hobbs, spoke on behalf of the many surgeons who trained in Bristol and owed so much to Joe's teaching and encouragement over so many years.

R.C.N.W.

**Outside Activities**

From student days onwards I have been impressed by the sporting and cultural activities which our profession manages to enjoy. I think they play an important part in preserving our mental and physical health. When I first came to Bristol there was a thriving Medical Dramatic Society with leading lights such as Sidney Curwen and John Naish. Unfortunately, this has faded out, but there is the thriving Southmead Orchestral Society run by our editor. This has given great pleasure and relaxation not only to the participants, but also to audiences in Wells and elsewhere.

The clergy–doctors group and the Bristol Medico Legal Society have been successful ventures which link with other professions. The major hospitals have their tennis and cricket clubs which often give an opportunity for friendship with colleagues in non-medical disciplines. The Hey Groves Golfing Society has gone from strength to strength so that matches are now played in Bath and Taunton as well as Bristol. Instead of being exclusively B.R.I. and B.G.H. based, the golfers now come from all the hospitals, including the dentists and general practitioners. Skiing is a popular sport in our profession, and each year large numbers of all ages travel to the Alps to enjoy the exhilaration of speed, beautiful scenery and good companionship spiced with an element of danger. Since I broke my leg 16 years ago I have been an ardent ski-bobber, and would be glad to introduce anyone to this equally exciting sport.

I believe these activities—and there are many more—help us to keep friends within our profession and to foster them in related professions. They help to reduce misunderstandings and enmity. I wish we could revive some of the events such as nurses prizegiving, fancy dress dances, hospital pantomimes and sisters dinners which helped our relationships with the nursing profession. I sense a real danger at present of separatism, which cannot be to the benefit of the most important group of all—our patients.

H.G.M.

**Doctors and Nurses**

The other day a nurse manager asked me what I thought about nurses in the community looking after their patients in hospital beds. She was talking about the care of elderly patients who—in the opinion of the district nurse—would be better managed in a hospital bed. After initial surprise my reply was distinctly negative. The question posed by this nurse though is indicative of a dramatic change in the relationship between nurses and doctors.

The Salmon management changes in nursing during the past decade or so have established within the hospital service a strong nursing profession. This profession manages itself—has its own personnel service and its own budget. Consultants no longer have a say in which nurse manages 'their' patients.

Following the reorganisations in the N.H.S. this structured nursing profession is now extended into the community. The relationship between the G.P. and the district nurse and health visitor that was developed by far-sighted medical officers of health in the sixties and seventies is slowly being eroded by the management structure in the new district authorities. Decisions of a medical nature are now being made by senior nursing management which adversely influence the relationship between a G.P. and the district nurse. Let me give two recent examples of the difficulties that have arisen within our health district.

Nurses should not give immunisations without a doctor being present. On the surface this is a very reasonable statement but the implications of this are very far reaching and no nurse can challenge this edict. I was asked by a consultant to arrange for weekly immunoglobulin injections to be given to a child with congenital deficiency of this—the nurses had to refuse to give it until senior management could be persuaded with considerable difficulty to make an exception in this case. The nurse has had to refuse to give an influenza immunisation to a patient with severe chronic obstructive airways disease who cannot leave his home for the same reason—despite the fact that anaphallactic reactions to this are as rare or rarer than reactions following penicillin injections. These, incidentally, can still be given by a nurse without a doctor holding her hand!

Nurse managers still maintain that they must appoint and decide on the numbers of nurses working in health centre treatment rooms despite the fact that the G.P.s pay the nurses from their own remuneration.

There is a suggestion that following the creation of independent Family Practitioner Committees that a new community nursing structure should be developed based on it and separated again from the district health authority. My recent experience would welcome this change—hospital nursing experience is insufficient to teach nurse managers what to do in the community. Wouldn't it be wonderful if we could revert to the old attitudes of trust that used to exist where a medical decision was carried out without recourse to dogma created by someone with a limited horizon.

M.J.W.

**Roles Reversed**

I had been quietly congratulating myself on how well I had been feeling, all things considered, during the year and had gone to bed as normal. I woke up earlier in the night feeling quite well and then went off to sleep again. About 4 a.m. I was wakened by an ache down the left side of the chest and inner side of the left arm. I felt terribly cold and realised what had happened. I woke my wife and asked her to check my blood pressure (sphygmomanometer was at hand). She was unable to obtain a reading so phoned the general practitioner. One of his partners arrived in what seemed a very short time and quickly appraised the situation. After establishing that a bed was available in the Coronary Care Unit of the nearest Hospital (Ham Green) phoned for an ambulance. He gave me Trinitrin tablets which helped. In an incredibly short time the ambulance arrived, I knew the two ambulancemen quite well and was grateful for their efficiency and reassurance. At that time in the morning there was little on the roads and they were able to travel at a speed with which my wife, who was following in the car, found difficulty in keeping up. On reaching the hospital, the last thing I think I can remember was being wheeled into the ward housing the Coronary Care Unit. I learned later that as I was being put into bed I 'arrested'. Fortunately the doctors who were on duty that night were waiting for me and started resuscitation immediately. The next thing I remember was waking up with an oxygen mask on my face and everyone reassuring me that I would be alright. Apart from some discomfort in the chest from the resuscitation (cardiac massage and defibrillation) – a small price to pay as it saved my life – I felt 'not too bad', though movement was restricted by a drip in one arm and a cannula in the other.

I am sure my recovery was aided by support from my wife who sat initially by my bed quietly for long periods and by my children, who kept popping in, and by visits from staff and friends and from all the 'get well' cards. I shall always be grateful for the skill and speed with which I was treated and the care shown by everyone, and will continue to believe in the value of Coronary Care without which this article would not have been written.

